# Comparison of Antibacterial Adhesion When Salivary Pellicle Is Coated on Both Poly(2-hydroxyethyl-methacrylate)- and Polyethylene-glycol-methacrylate-grafted Poly(methyl methacrylate)

**DOI:** 10.3390/ijms19092764

**Published:** 2018-09-14

**Authors:** Bor-Shiunn Lee, Yu-Jia Chen, Ta-Chin Wei, Tien-Li Ma, Che-Chen Chang

**Affiliations:** 1 Graduate Institute of Oral Biology, School of Dentistry, National Taiwan University and National Taiwan University Hospital, No.1, Changde St., Jhongjheng District, Taipei 100, Taiwan; leebs@ntu.edu.tw (B.-S.L.); bmjdi10607@gmail.com (Y.-J.C.); lily4714734@yahoo.com.tw (T.-L.M.); 2 Department of Chemical Engineering, Chung Yuan Christian University, 200, Chung Pei Rd., Chung Li 320, Taiwan; tcwei@cycu.edu.tw; 3 Department of Chemistry, National Taiwan University, No. 1, Sec. 4, Roosevelt Road, Taipei 10617, Taiwan

**Keywords:** anti-adhesion, antibacterial adhesion, protein adsorption, pellicle coating, dentistry, orthodontic, biocompatibility, serial dilution spotting, prostheses

## Abstract

Although poly(2-hydroxyethyl methacrylate) (pHEMA) and polyethylene glycol methacrylate (PEGMA) have been demonstrated to inhibit bacterial adhesion, no study has compared antibacterial adhesion when salivary pellicle is coated on polymethyl methacrylate (PMMA) grafted with pHEMA and on PMMA grafted with PEGMA. In this study, PMMA discs were fabricated from a commercial orthodontic acrylic resin system (Ortho-Jet). Attenuated total reflection-Fourier transform infrared spectra taken before and after grafting confirmed that pHEMA and PEGMA were successfully grafted on PMMA. Contact angle measurements revealed PMMA-pHEMA to be the most hydrophilic, followed by PMMA-PEGMA, and then by PMMA. Zeta potential analysis revealed the most negative surface charges on PMMA-PEGMA, followed by PMMA-pHEMA, and then by PMMA. Confocal laser scanning microscopy showed green fluorescence in the background, indicating images that influenced the accuracy of the quantification of live bacteria. Both the optical density value measured at 600 nm and single plate-serial dilution spotting showed that pHEMA was more effective than PEGMA against *Escherichia coli* and *Streptococcus mutans*, although the difference was not significant. Therefore, the grafting of pHEMA and PEGMA separately on PMMA is effective against bacterial adhesion, even after the grafted PMMA were coated with salivary pellicle. Surface hydrophilicity, bactericidality, and Coulomb repulsion between the negatively charged bacteria and the grafted surface contributed to the effectiveness.

## 1. Introduction

Persistent biofilm formation on medical devices such as implants, catheters, and ureteral stents can cause chronic infection and, ultimately, often leads to replacement. Therefore, the development of a new strategy or materials to combat biofilm formation has been a critical topic of research. Numerous materials with antibacterial properties have been proposed. For example, silver is a broad-spectrum antimicrobial metal against Gram-positive and Gram-negative bacteria [1]. Its incorporation as silver zeolite or silver nanoparticles has been described for use in medical devices [2]. Other inorganic chemicals demonstrating antibiotic activity in the form of nanoparticles include ZnO [3], TiO_2_ [4], Cu and CuO [5].

In dentistry, biofilm formation on material surfaces or dental hard substances can cause oral diseases such as peri-implantitis, caries, gingivitis, and periodontitis [6,7]. One of the widely used materials in the oral cavity is methacrylate polymer. Chlorhexidine has been added to the polymer matrix for controlled release to prevent bacterial adhesion to methacrylate material [8]. Other approaches for inhibiting adhesion include the incorporation of 12-methacryloyloxydodecylpyridinium bromide and quaternary ammonium methacryloxy silicate (QAMS) [9,10] in acrylic-based resin materials, which demonstrates bactericidal activity against oral bacteria, thus preventing bacteria- and fungus-induced stomatitis. In addition to the antibacterial effect, results have also shown that QAMS incorporation does not affect the flexural strength or modulus [11].

The factors affecting bacterial adhesion on biomaterial surfaces include surface morphometry, physico-chemical properties, environmental conditions, and pathogens [12]. The adjustment and optimization of surface morphometry and physico-chemical properties may be achieved through surface coating or grafting. A previous study reported that a 2-hydroxyethyl methacrylate (HEMA) coating on cellulose acetate increased surface hydrophilicity and improved resistance to seawater microbial biofouling [13]. Similar results were obtained for polyHEMA (pHEMA) grafted on gold, which inhibited adhesion of *Cytophaga lytica* in seawater [14]. The other molecule frequently used to increase surface hydrophilicity is polyethylene glycol (PEG). Grafting PEG methacrylate (PEGMA) on the surface of polypyrrole can reduce protein adsorption and bacterial adhesion [15]. Studies on the relationship between surface roughness and bacterial adhesion have shown that increased roughness causes increased adhesion of *Staphylococcus epidermidis* and *Pseudomonas aeruginosa* on the surfaces of contact lenses [16] and rigid gas permeable lenses [17], respectively.

Among methacrylate polymers, polymethyl methacrylate (PMMA) is generally used in dentistry for denture base materials, maxillofacial prostheses, temporary restoration, and orthodontic appliances. However, no comparative studies have been conducted on pHEMA and PEGMA coatings on PMMA for antibacterial adhesion. This study comparatively investigated the antibacterial effect of PMMA grafted with pHEMA (termed PMMA-pHEMA hereafter) or PEGMA (termed PMMA-PEGMA hereafter) against adhesion of two bacteria, *Streptococcus mutans* and *Escherichia coli*. These are common bacteria found in the oral cavity that have been used as model organisms to represent Gram-positive and Gram-negative bacteria, respectively [18,19]. In particular, the antibacterial activity of PMMA-pHEMA and PMMA-PEGMA coated with salivary pellicle was studied. The salivary pellicle is always present on the surfaces of teeth and oral apparatus. However, previous studies have not mimicked this condition before bacterial contact. The possible effects of the presence of salivary pellicle on the adherence of bacteria to the pHEMA- or PEGMA-grafted materials are still unknown. The presence of salivary pellicle on modified titanium surfaces (machined, acid-etched, and acid-etched and blasted) may result in negatively charged surfaces that attract calcium ions and facilitate the adhesion of *S. mutans* and *Fusobacterium nucleatum* to the surfaces [20]. However, studies of the adherence of S. mutans to hydroxyapatite precoated with whole saliva, with or without the presence of the antibacterial agent Lysozyme, showed a significant reduction in the adherence [21,22]. The aim of this study is to compare the antibacterial adhesion of pHEMA- and PEGMA-grafted PMMA when salivary pellicle is coated on the grafted PMMA.

## 2. Results

**Figure 1:**
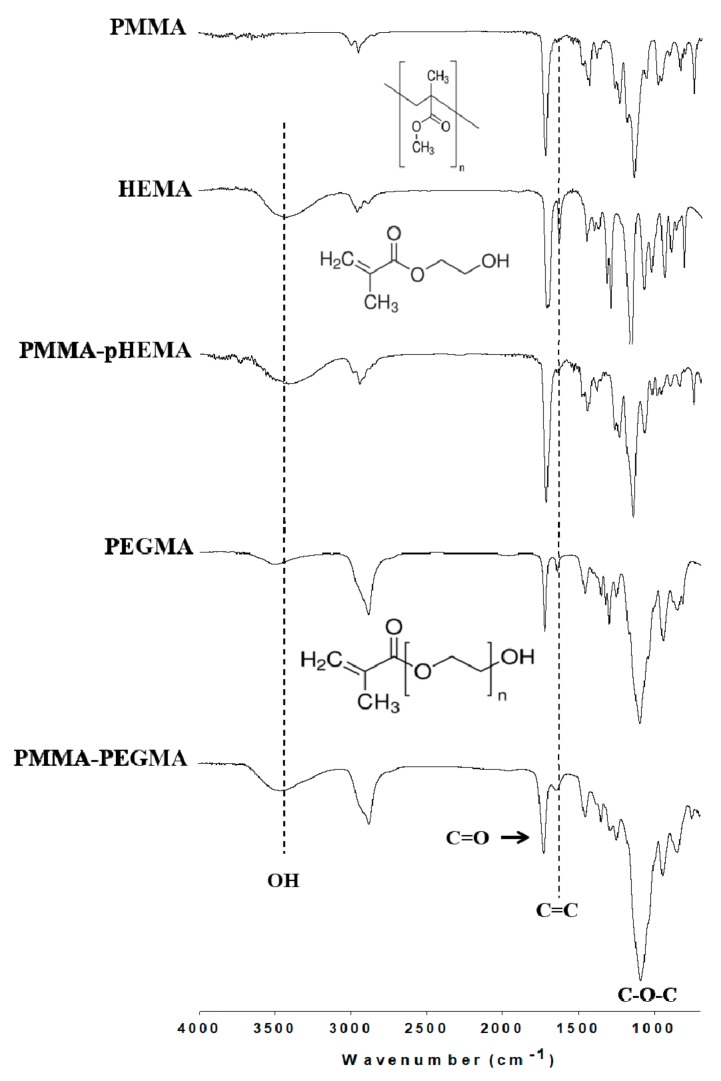
Fourier transform infrared (FTIR) spectra of polymethyl methacrylate (PMMA), 2-hydroxyethyl methacrylate (HEMA), PMMA-poly(2-hydroxyethyl methacrylate) (pHEMA), polyethylene glycol methacrylate (PEGMA), and PMMA-PEGMA.

Figure 1 shows the Fourier transform infrared (FTIR) spectra of PMMA, HEMA, PMMA-pHEMA, PEGMA, and PMMA-PEGMA. The characteristic peaks of PMMA included C=O (1720 cm^−1^) and ester OC–O–C (1165 cm^−1^) stretching vibrations [23]. In addition to the C=O and OC–O–C characteristic peaks, HEMA contained OH (3450 cm^−1^) and C=C (1635 cm^−1^) stretching vibrations [24]. The C=C stretching vibration was not found in PMMA-pHEMA, indicating that almost all HEMA monomers on PMMA were polymerized. In addition to the characteristic peaks of OH, C=O, C=C, and OC–O–C stretching vibrations, the PEGMA spectrum exhibited a strong CC–O–C (approximately 1100 cm^−1^) stretching band [25] caused by the presence of a long glycol chain in the PEGMA molecule. Because the absorption peak of CC–O–C shifts slightly to higher wavenumbers with increasing glycol units in the molecular chain [26], the observed broad band indicated that PEGMA had different lengths of glycol chains. After PEGMA was grafted on PMMA, the characteristic peaks and the CC–O–C band profile of the PMMA-PEGMA spectrum were similar to those of the PEGMA spectrum.

**Figure 2:**
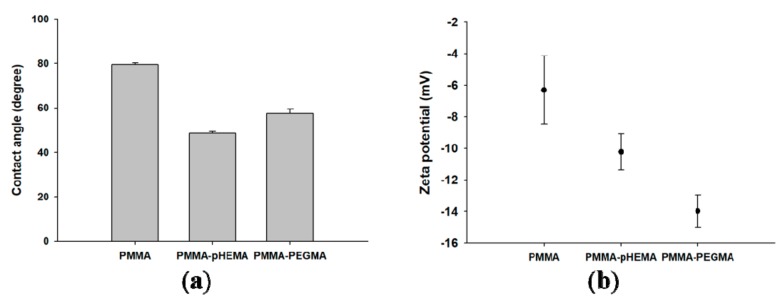
(**a**) Contact angles expressed in degrees and (**b**) zeta potentials (mV) of PMMA, PMMA-pHEMA, and PMMA-PEGMA.

The average contact angles of PMMA, PMMA-pHEMA, and PMMA-PEGMA were 79.56° ± 0.71°, 48.65° ± 0.75°, and 57.41° ± 2.14°, respectively (Figure 2a). PMMA-pHEMA was the most hydrophilic, followed by PMMA-PEGMA and PMMA. The zeta potentials (mV) of the salivary-pellicle-covered PMMA, PMMA-pHEMA, and PMMA-PEGMA were −6.31 ± 2.16, −10.23 ± 1.15, and −13.98 ± 1.03, respectively (Figure 2b). PMMA-PEGMA exhibited the most negative surface charge, followed by PMMA-pHEMA and PMMA.

**Figure 3:**
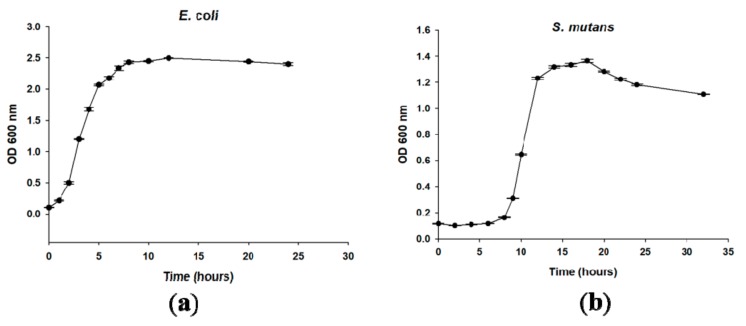
Bacterial growth curves of (**a**) *E. coli* and (**b**) *S. mutans* with an initial concentration of 0.1 OD_600_.

**Figure 4:**
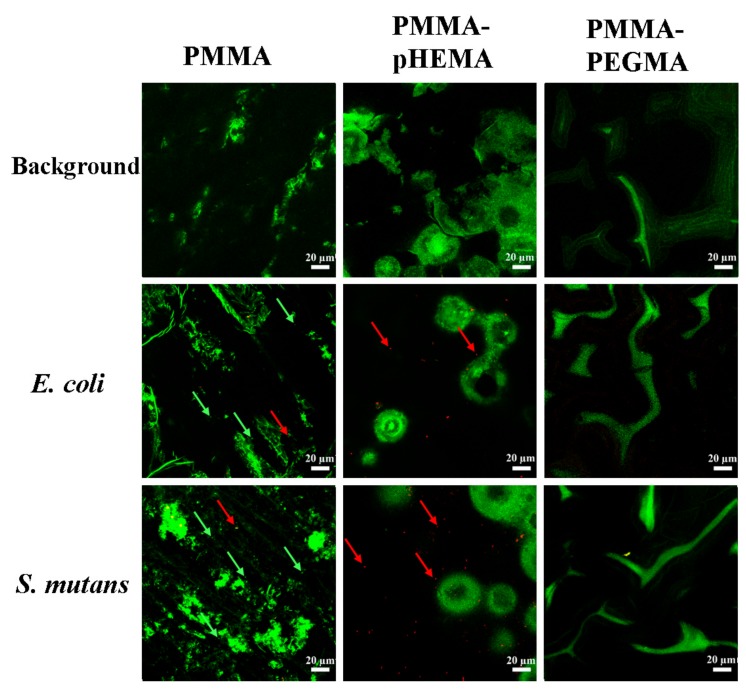
Confocal laser scanning microscope (CLSM) images of PMMA, PMMA-pHEMA, and PMMA-PEGMA discs without bacterial inoculation (**top row**) and after 2 h of *E. coli* (**middle row**) or *S. mutans* (**bottom row**) adhesion. Green arrows selectively indicate the presence of live bacteria (in green), whereas red arrows selectively indicate the presence of dead bacteria (in red).

**Figure 5:**
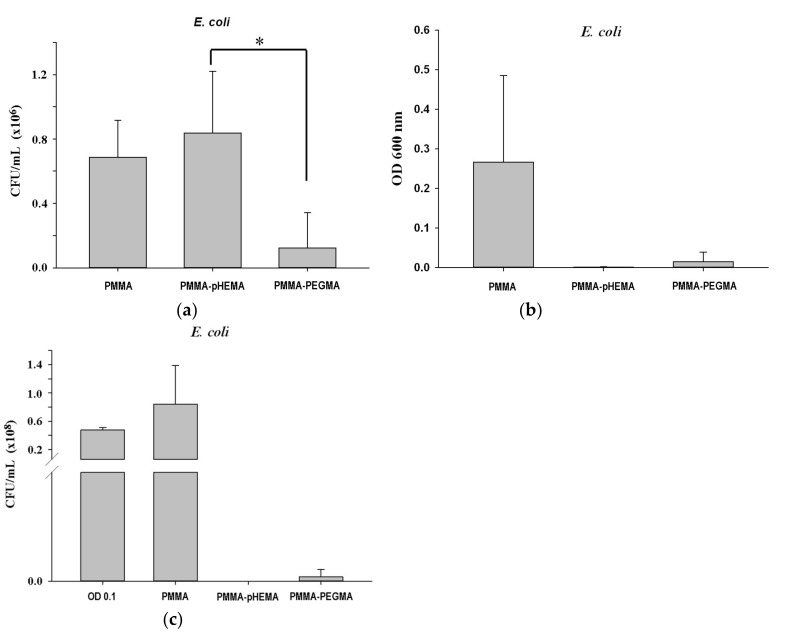
(**a**) colony-forming units (CFU)/mL values of *E. coli* in washed phosphate buffered saline (PBS) from the first incubation after being cultured on tryptic soy agar (TSA) using SP-SDS. The (**b**) OD_600_ and (**c**) CFU/mL values of *E. coli* in culture medium after the second incubation for 3 h. * *p* < 0.05.

**Figure 6:**
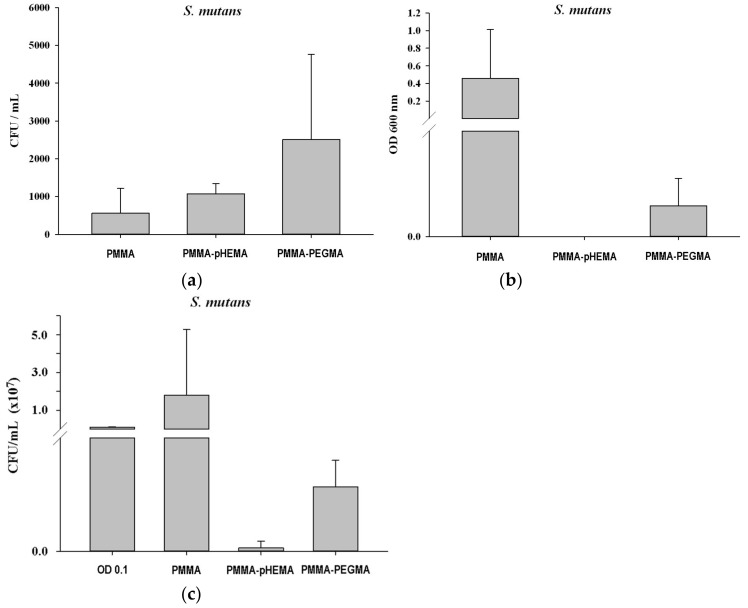
(**a**) CFU/mL values of *S. mutans* in washed PBS from the first incubation after being cultured on TSA using SP-SDS. The (**b**) OD_600_ and (**c**) CFU/mL values of *S. mutans* in culture medium after the second incubation for 13 h.

**Figure 7:**
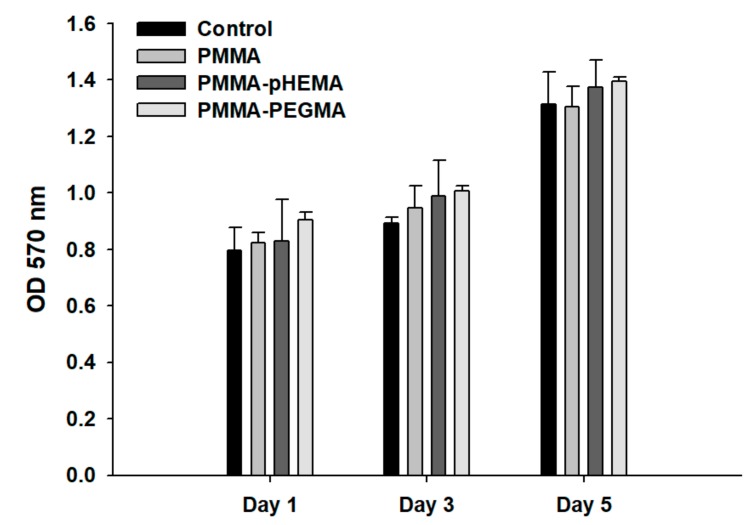
MTT results of the control, PMMA, PMMA-pHEMA, and PMMA-PEGMA. All 3 PMMA-related materials were considered biocompatible because the OD_570_ values were similar to the control group.

Figure 3 shows the bacterial growth curves of *E. coli* and *S. mutans* with the initial concentration at 0.1 OD_600_. For *E. coli*, the timing of the lag, log, and stationary phases was approximately 0–2 h, 2–8.5 h, and 8.5–24 h, respectively. For *S. mutans*, the timing of the lag, log, stationary, and death phases was approximately 0–8 h, 8–13 h, 13–18 h, and 18–32 h, respectively. Figure 4 shows confocal laser scanning microscope (CLSM) images of PMMA, PMMA-pHEMA, and PMMA-PEGMA discs without bacterial inoculation and after 2 h of *E. coli* or *S. mutans* adhesion. Because green fluorescence was observed in the background of 3 materials (top row), it was difficult to differentiate the live bacteria from the background in the evaluation of *E. coli* or *S. mutans* adhesion. Both *E. coli* and *S. mutan* were scarce in the PMMA-PEGMA group under CLSM observation. By contrast, numerous green and red spots were found in the PMMA and PMMA-pHEMA groups, respectively. Figure 5a shows the amount of *E. coli* obtained from washed phosphate buffered saline (PBS) and cultured on tryptic soy agar (TSA) using SP-SDS. The PMMA-pHEMA group exhibited significantly higher *E. coli* than the PMMA-PEGMA group (*p* < 0.05). Figure 5b,c show the OD_600_ and colony-forming units (CFU)/mL values, respectively, of the culture medium after the washed materials exposed to *E. coli* were incubated again for 3 h. The statistical analysis of both results is shown in Table 1. Figure 5 and Table 1 show that PMMA-pHEMA and PMMA-PEGMA exhibited significantly lower amounts of bacteria than PMMA. Figure 6a shows the amount of *S. mutans* obtained from washed PBS and cultured on TSA using SP-SDS. The PMMA-PEGMA group exhibited a greater amount of *S. mutans* than the PMMA-pHEMA and PMMA groups. Figure 6b,c show the OD_600_ and CFU/mL values, respectively, of the culture medium after the washed materials exposed to *S. mutans* were incubated again for 13 h. The statistical analysis of both results is also shown in Table 1. Figure 6 and Table 1 show that PMMA-pHEMA and PMMA-PEGMA exhibited significantly lower OD_600_ values than PMMA (*p* < 0.05). In addition, the antibacterial adhesion of PMMA-pHEMA was better than that of PMMA-PEGMA. Figure 7 shows the MTT results of the tested materials. All 3 materials were considered biocompatible because the OD_570_ values were similar to the control group.

## 3. Discussion

Adhesion of oral bacteria to PMMA is a complex process affected by variables including surface roughness, charge, and hydrophilicity [6]. Increased surface roughness enhances bacterial adhesion because of the increase in contact area [27]. Because the PMMA discs were smoother than the PMMA-pHEMA and PMMA-PEGMA discs, all PMMA discs were roughened to similar roughness as the other 2 materials to exclude the effect of roughness on bacterial adhesion. Materials with Ra values smaller than 0.2 μm have been reported to be effective in reducing bacterial adhesion on abutment surfaces [28]. The 3 tested materials had Ra values of approximately 0.5 μm, indicating that bacterial adhesion was not prevented by higher roughness.

Surface grafting of PMMA with pHEMA was achieved through thermal polymerization of HEMA monomers in the presence of benzoyl peroxide, which acted as a thermal free radical initiator. The FTIR spectra of PMMA-pHEMA exhibited a broad absorption band of OH stretching but did not demonstrate C=C stretching vibration (Figure 1). This supports the notion that pHEMA was successfully grafted onto PMMA and almost all HEMA monomers on PMMA were thermally polymerized. The resulting pHEMA-PMMA exhibited smaller contact angles than PMMA (Figure 2a) because of the higher hydrophilicity of the glycolic hydroxyl groups on pHEMA-PMMA than the methoxy groups on PMMA.

PEGMA was grafted onto PMMA using a different method. In the pilot study, the thermal polymerization method failed to effectively modify the PMMA surface with PEGMA, despite HEMA and PEGMA both having a methacrylate skeleton and containing ethylene glycol (EG) side chains, with HEMA having one EG unit in the chain and PEGMA having several. The failure was possibly caused by the presence of the long PEG chain in PEGMA, which excluded effective attack of radical initiators to the carbon–carbon double bond in the methacrylate part of PEGMA or, if radical initiators incidentally transferred radicals to PEGMA, effective reaction of the formed PEGMA radicals with PMMA. Therefore, atmospheric pressure plasma was used to fabricate PMMA-PEGMA. However, HEMA monomers could not be polymerized through plasma treatment to form PMMA-pHEMA.

The FTIR spectra of PMMA-PEGMA exhibited the CC–O–C stretching vibration band with a bandwidth and band profile similar to that of PEGMA (Figure 1). Because the longer glycol chains of PEGMA lead to the absorption bands of the CC–O–C stretching vibration with higher wavenumbers [26], the similarity indicated that the fabrication of PMMA-PEGMA using atmospheric pressure plasma did not substantially favor PEGMA with shorter glycol chains for grafting. Instead, as suggested by the similar bandwidths and band profiles, the fabricated PMMA-PEGMA exhibited glycol chains with a chain length distribution resembling that of PEGMA. The distribution, and thus the difference in length of the glycol chains of PEGMA on PMMA, however, caused the alkoxy portion of the chains to be exposed on the PMMA-PEGMA surface. By contrast, the PMMA-pHEMA surface was covered with hydroxyl groups, which were situated at the same short height (i.e., equivalent to one EG unit) above the polymeric C–C backbone of pHEMA. Because alkoxy is less hydrophilic than hydroxyl, the PMMA-PEGMA surface yielded larger contact angles than the PMMA-pHEMA surface (Figure 2a), despite the angles still being smaller than that measured on PMMA because of the hydrophobic property of the methyl ester group of PMMA.

The resistance of the salivary-pellicle-covered surface of the grafted PMMA, created through incubation in pooled sterile saliva, against bacterial adhesion was studied. The population and survival of bacteria on the salivary-pellicle-covered surface were firstly analyzed using CLSM, which is a tool frequently used to examine live/dead bacteria and the depth of a biofilm. CLSM images of PMMA, PMMA-pHEMA, and PMMA-PEGMA discs, without bacterial inoculation, exhibited a background of green fluorescence (top row of Figure 4). After a 2-h inoculation with a drop of bacterial suspension on the sample for adhesion followed by washing with sterile PBS for removal of non-adherent bacteria, dead bacteria (revealed as red spots) were observed in the CLSM images of PMMA-pHEMA (bottom two rows in the middle column of Figure 4). Conversely, dead bacteria were almost absent in the PMMA-PEGMA group under CLSM observation (bottom two images of the right column, Figure 4). In both cases, the green spot indicating live bacteria, the intensity of which is typically greater than that of the background, was not distinguishable from the background in this study. The accuracy of the quantification of live bacteria, and thus the determination of the antibacterial property of the sample, may have been severely impaired by the use of CLSM images.

Consequently, the PBS collected from washing the salivary-pellicle-covered sample surface after the 2-h bacterial inoculation for adhesion (termed the first incubation hereafter) was cultured on TSA using SP-SDS to calculate its CFU/mL values. The amount of the unattached, live bacteria remaining on the PMMA-pHEMA surface before PBS washing could thus be estimated from the calculated CFU values. Depending on the bacteria tested, the amount on PMMA-pHEMA may be significantly larger (for *E. coli*, Figure 5a) or smaller (for *S. mutans*, Figure 6a) than that on PMMA-PEGMA.

In addition, to quantify the live bacteria attached to the sample after washing, the washed sample was incubated again (termed the second incubation hereafter) in a new sterile tryptic soy broth (TSB) for 3 h for *E. coli* and 13 h for *S. mutans*. The incubation time was selected based on the log phase in which the bacteria demonstrated rapid growth (Figure 3). The OD_600_ and CFU/mL values of the culture medium were measured after the second incubation. Higher OD_600_ and CFU/mL values of the culture medium after the second incubation were expected if more live bacteria remained attached to the sample surface after the PBS wash following the first incubation. Both *E. coli* (Figure 5b,c) and *S. mutans* (Figure 6b,c) showed lower OD_600_ and CFU/mL values on PMMA-pHEMA and PMMA-PEGMA than on PMMA, indicating that, after PBS washing in the first incubation, far fewer live bacteria were attached to the surface of the grafted PMMA than to the surface of the ungrafted PMMA. PMMA-pHEMA and PMMA-PEGMA thus displayed better antibacterial adhesion activity than PMMA even after their surfaces were covered with salivary pellicle.

The observed smaller amounts of live bacteria attached to the grafted PMMA surfaces may have been caused partially by the repulsive interaction of the surface with the bacteria. Both *E. coli* and *S. mutans* surfaces are negatively charged in water. A huge percentage of the cell wall of most Gram-positive bacteria is composed of teichoic acids, which are anionic because of the presence of phosphate in their structure [29]. For Gram-negative bacteria, the outer leaflet of their membrane is composed principally of lipopolysaccharide, which imparts a strongly negative charge to the cell surface [30]. Nevertheless, the nature of the negative charge on the *E. coli* and *S. mutans* surfaces is still being investigated. Zeta potential measurements showed that the pHEMA- and PEGMA-grafted PMMA surfaces were also negatively charged (Figure 2b). The Coulomb repulsion between the negatively charged bacteria and the grafted surface thus significantly reduced the interaction between the bacteria, brought in by fluid hydrodynamics, and the grafted surface. The reduction led to far fewer live bacteria with the opportunity to attach directly to the surfaces of the grafted PMMA than the ungrafted PMMA (Figure 5b,c and Figure 6b,c).

Surface hydrophilicity also affected the number of live bacteria attached to the sample surface. Both PMMA-pHEMA and PMMA-PEGMA were hydrophilic. As indicated by the larger average contact angles of PMMA-PEGMA than PMMA-pHEMA (Figure 2a), PMMA-PEGMA was less hydrophilic than PMMA-pHEMA because of its longer PEG side chains than the side chain of pHEMA. Bacterial cells with hydrophobic walls were thus less repulsed by PMMA-PEGMA than PMMA-pHEMA. In addition, *E. coli* cells use flagella to attach to surfaces [30] and *S. mutans* cells produce exopolysaccharides for their colonization on substrates [31]. A denser layer of water was formed on PMMA-pHEMA than on PMMA-PEGMA, which could have led to greater weakening of the interaction between bacteria and the material. Higher optical density (OD) and CFU/mL values of the culture medium from the second incubation for both *E. coli* (Figure 5b,c) and *S. mutans* (Figure 6b,c) were thus obtained for PMMA-PEGMA than for PMMA-pHEMA.

The substantially smaller number of live bacteria on the grafted PMMA than the ungrafted PMMA and the larger number of live bacteria on PMMA-PEGMA than PMMA-pHEMA, both observed in the culture medium from the second incubation, were not observed in the PBS wash from the first incubation. Comparison of the number of live bacteria on PMMA-pHEMA with that on PMMA-PEGMA measured for *E. coli* revealed a disparity between the washed PBS in the first incubation (Figure 5a) and the culture medium of the second incubation (Figure 5b,c). As discussed in Appendix A, this disparity revealed the grafted PMMA surface to be bactericidal because of the difference in cell wall structure between Gram-positive and Gram-negative bacteria and the possible formation of bacteria sandwiched between weakly bound bi-layers of wall fragments, a result of cell lysis generated by the EG side chains, which acted as biosurfactants.

PMMA is toxic to mammalian cells because of its leaching of unreacted methyl methacrylate (MMA) [32]. In this study, cell viability was examined using MTT assays and the results showed no significant difference between PMMA and the control (Figure 7). The reason may have been that PMMA discs were placed in Aquapres at 1.5 kg/cm^2^ for 1 h to remove most of the unreacted MMA. PMMA-pHEMA and PMMA-PEGMA were also biocompatible because their cell viability was similar to the control, suggesting that leaching of toxic components was negligible. This may be an advantage of grafting compared with incorporation of antimicrobial agents throughout the bulk polymer network.

## 4. Materials and Methods

**Figure 8:**
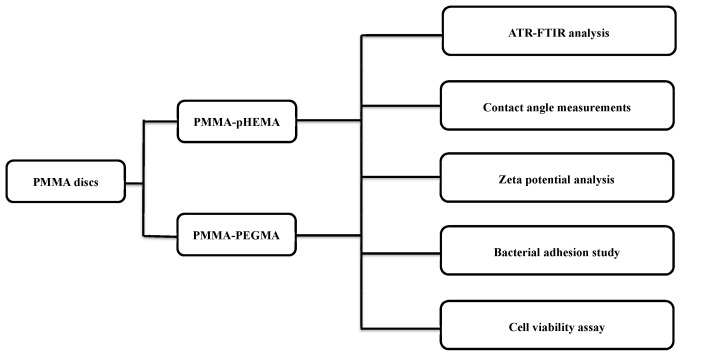
Flow diagram of this study.

Figure 8 shows a flow diagram which presents the grafting process, applied characterization techniques, and tested groups for the antibacterial adhesion of the grafted PMMA. The detail is discussed below.

### 4.1. Materials

HEMA (99%, MW = 130.14) and PEGMA (MW = 500) of analytical grade were purchased from Sigma-Aldrich (Milwaukee, WI, USA). PMMA discs were fabricated from a commercially available, auto-polymerizing, methyl methacrylate/poly(methyl methacrylate) (MMA/PMMA) orthodontic acrylic resin system (Ortho-Jet; Lang Dental Manufacturing Co. Inc., Wheeling, IL, USA). MMA monomers (0.1 mL) were mixed with 0.1 g of PMMA powder and the mixture was then placed in stainless steel molds to produce 5-mm-diameter and 1-mm-thick discs. After gelling, PMMA discs were placed in Aquapres (Lang Dental Manufacturing Co. Inc., Wheeling, IL, USA) at 1.5 kg/cm^2^ for 1 h.

### 4.2. pHEMA and PEGMA Grafting on PMMA

All PMMA discs underwent ultrasonic cleaning in 75% ethanol and deionized water for 5 min. For pHEMA modification, PMMA discs were immersed in 30 mL of 0.05 M benzoyl peroxide (≥97% purity, Alfa Aesar, Ward Hill, MA, USA) and 99.5 wt.% ethanol solution at 25 °C for 30 min under nitrogen. Subsequently, 30 mL of 1.5 M HEMA in an aqueous solution was added slowly at 65 °C and the reaction between HEMA and the peroxide-modified PMMA discs occurred for 2 h under a stream of nitrogen. The pHEMA-modified discs were then washed several times with deionized water and methanol to remove unreacted monomers before they were placed in a vacuum drying oven at 50 °C for 48 h [33]. For PEGMA modification, 5 μL of PEGMA was dropped onto each disc at room temperature and the reaction between PEGMA and PMMA occurred for 20 min. After the reaction, the disc surface was activated for 15 min by an atmospheric pressure plasma jet generated by nitrogen gas with a flow rate of 40 standard liters per minute and an input power of 500 W (scan number = 240 times). All PEGMA-modified discs underwent ultrasonic cleaning in isopropanol for 30 min. Because the surfaces of PMMA-pHEMA and PMMA-PEGMA were rougher than those of PMMA, the PMMA samples were roughened with 240 grit SiC paper for approximately 2 min using Buehler Ecomet 3 Polisher/Grinder (Buehler Ltd., Lake Bluff, IL, USA). A surface profilometer (Surfcorder ET 200, Kosaka, Japan) was used to confirm that the PMMA samples after roughening had similar surface roughness (Ra, the arithmetic average of the absolute deviations of the profile heights and valleys from the mean line) to the modified PMMA. The tracing diamond tip was 2 μm with a tracing speed of 0.2 m/s, force of 200 μN, tracing length of 1 mm, and cutoff value of 0.8 mm. Three tracings were performed at different locations on the surface of each specimen (*n* = 5). The Ra values of PMMA, PMMA-pHEMA, and PMMA-PEGMA were 0.50 ± 0.02 μm, 0.55 ± 0.09 μm, and 0.50 ± 0.15 μm, respectively.

### 4.3. Attenuated Total Reflection (ATR)-FTIR Analysis

The surfaces of PMMA, PMMA-pHEMA, and PMMA-PEGMA were examined using ATR-FTIR (FTIR-4200, Jasco International Co., Ltd., Tokyo, Japan). FTIR spectra were recorded by pressing the samples against the ZnSe ATR crystal at a slow scan rate and normal slit width. The wavelength used was in the range of 4000–650 cm^−1^.

### 4.4. Contact Angle Measurements

Static contact angles were measured with the sessile drop method of water drops at room temperature using FTA125 (First Ten Ångstroms, Inc., Portsmouth, VA, USA). Each specimen was mounted at a height sufficiently close to the delivery needle of a syringe, and water droplets (approximately 3 μL) were delivered to different points of each specimen. An image was then captured, and the contact angle was calculated using the tangent method.

### 4.5. Zeta Potential Analysis

PMMA, PMMA-pHEMA, and PMMA-PEGMA samples (44 mm × 25 mm) were prepared and sterilized in 75% ethanol before being placed under ultraviolet light irradiation overnight. Saliva (1 mL) was dropped on each sample. The zeta potential was measured using the SurPASS Electro-kinetic Analyzer (Anton-Paar KG, Graz, Austria) by clipping the sample to a flat-plate measuring cell. The target ramp pressure was 500 mbar. Samples were placed in a NaCl electrolyte solution, instead of the bacterial suspension, to avoid contamination from bacteria on the instrument. Through the addition of NaOH or HCl, the pH of the solution was adjusted to pH 7, which was equivalent to that of the bacterial suspension, before the streaming potential was measured at a target ramp pressure of 500 mbar. To measure the effect of the ionic strength of the solution, 0.1, 1, 10, and 100 mM NaCl solutions were used. Zeta potentials were calculated from the measured streaming potentials using the Fairbrother–Mastin (F–M) equation [34].

### 4.6. Bacterial Culture

Gram-positive *S. mutans* and Gram-negative *E. coli* stored at −80 °C were separately cultured on TSA; BD Biosciences, Franklin Lakes, NJ, USA) at 37 °C overnight. A strain of a single colony on TSA was then cultured in a 10 mL TSB; BD Biosciences) at 37 °C with an orbital shaker incubator at 220 rpm for 16 h. After 16 h of culture, the *S. mutans* and *E. coli* strains were harvested by centrifugation at 3000 rpm for 10 min. The resultant bacterial pellet was washed 3 times with sterile PBS and then adjusted to a concentration of 10^7^ CFU/mL (OD_600_ = 0.1) before use.

### 4.7. Bacterial Growth Curve

The strains of *S. mutans* and *E. coli* were cultured separately for 16 h in 3 mL of TSB from a single colony that grew on TSA. The TSB with bacterial suspension was then diluted 100 times and cultured in an Erlenmeyer flask at 37 °C with an orbital shaker incubator at 220 rpm. The OD_600_ value was measured for 32 h to obtain the bacterial growth curve.

### 4.8. Bacterial Adhesion Study

The samples were sterilized using 75% alcohol for 5 min and ultraviolet irradiation for 16 h. Pooled sterile human saliva was collected according to the protocol used by Gong et al. [10] and the samples were incubated in pooled sterile saliva for 1 h at 37 °C to create salivary pellicle on the surface. The bacterial suspension (OD_600_ = 0.1, 10 μL) was dropped onto the sample surfaces followed by incubation at 37 °C for 2 h (*n* = 10). The samples were then washed with sterile PBS 3 times to remove non-adherent bacteria. The washed PBS was collected and cultured on TSA using SP-SDS [35]. TSA was drawn to 6 sectors with the first and last dilution sectors marked. Aliquots (20 μL) from the 6 selected dilutions were applied as 10–12 micro-drops in each sector. Half samples in each group were prepared for examination using CLSM. The other half of the samples were transferred aseptically to new sterile TSB for 3 h (*E. coli*) and 13 h (*S. mutans*) at 37 °C. At the end of incubation, the samples with adherent bacteria were removed from the culture medium. The OD_600_ value for 0.9 mL of culture medium was measured and 0.1 mL of culture medium was cultured on TSA using SP-SDS. The samples for examination using confocal laser scanning microscopy were dyed using a LIVE/DEAD BacLight Bacterial Viability Kit (Molecular Probes, Eugene, OR, USA) consisting of propidium iodide (PI) and SYTO^®^ 9. Green fluorescing SYTO^®^ 9 can label live bacteria and red fluorescing PI can label dead bacteria. Zeiss LSM 880 CLSM (Carl Zeiss Microscopy, Jena, Germany) at 40× magnification was used at excitation wavelengths of 488 nm and 561 nm for SYTO^®^ 9 and PI, respectively.

### 4.9. Cell Viability Assay

Primary cultures of human gingival fibroblasts (HGFs) were used. The trial was approved by the Research Ethics Committee of National Taiwan University Hospital (8 June 2011) and was registered with Case No. 201105080RC. Written informed consent was obtained from all participants collect gingival tissues. HGFs were cultured in Minimum Essential Medium alpha with 10% fetal bovine serum and 100 U/mL of antibiotics (penicillin–streptomycin–amphotericin; Sigma-Aldrich) at 37 °C in 5% CO_2_. The passage number was 8–12. The specimens in each group (*n* = 5) were placed on the bottoms of transwell inserts (Costar Transwell Permeable Supports, Corning, NY, USA; diameter 6.5 mm, pore size 3.0 mm). For comparison of the relative toxicities of the tested materials, the transwells were transferred into 24-well culture plates, which had been seeded with HGFs at 5 × 10^4^ cells per well and allowed to adhere overnight at 37 °C. Empty inserts served as a negative control group. After incubation for 1, 3, and 5 days, the cells in each well were incubated at 37 °C for 3 h with culture medium containing 100 μL of MTT solution. The medium was then aspirated and dimethyl sulfoxide (200 μL) was added to dissolve the reduced formazan crystals. The optical density (OD_570_) of the formazan solution was measured using a microplate reader (ELx 800, Biotek, Winooski, VT, USA).

### 4.10. Statistical Analysis

Statistical analysis was performed using the Statistical Package for the Social Sciences (version 22.0, Armonk, NY, USA). Tests were 2-tailed with the level of significance was set at 0.05. Descriptive statistics for continuous variables were calculated and reported as the mean ± standard deviation. The data were analyzed using one-way analysis of variance, and a later comparison between the groups was made using Fisher’s multiple comparison test.

## 5. Conclusions

Attachment of bacteria to medical devices is a severe problem because it leads to subsequent colonization, biofilm formation, and infection. Although major efforts have been focused on this problem, it remains a great challenge because the interaction of bacteria and materials varies from one kind of bacteria to another. In this study, modification of PMMA with pHEMA and PEGMA was achieved and FTIR-ATR analysis confirmed the presence of grafting on the PMMA surfaces. The adhesion of *E. coli* and *S. mutans* onto the modified surfaces was significantly inhibited, even after the surfaces were covered with salivary pellicle. Finally, the modified PMMA did not show cytotoxic effects in MTT assays using HGFs. Because prostheses or appliances made of PMMA must typically be worn for a prolonged period, the duration of antibacterial adhesion requires further study in the future.

## Figures and Tables

**Table 1:** Statistical analysis results of Figure 5 and Figure 6.

Paired Samples of Analysis	Test Statistics (*P*)	
	*E. coli*	*S. mutans*
(**a**)		
PMMA vs. PMMA-pHEMA	0.5252	0.1952
PMMA-pHEMA vs. PMMA-PEGMA	0.0174 *	0.4737
PMMA vs. PMMA-PEGMA	0.0118 *	0.2773
(**b**)		
PMMA vs. PMMA-pHEMA	0.0268 *	0.0404 ^#^
PMMA-pHEMA vs. PMMA-PEGMA	0.2481	0.1340
PMMA vs. PMMA-PEGMA	0.0340 *	0.0409 ^#^
(**c**)		
PMMA vs. PMMA-pHEMA	0.0219 *	0.0588
PMMA-pHEMA vs. PMMA-PEGMA	0.3160	0.1311
PMMA vs. PMMA-PEGMA	0.0221 *	0.0691

* Significant difference (*p* < 0.05). (**a**) CFU/mL values of *E. coli* in washed PBS from the first incubation after being cultured on TSA using SP-SDS. (**b**) The OD_600_ values of *E. coli* in culture medium after the second incubation for 3 h. (**c**) The CFU/mL values of *E. coli* in culture medium after the second incubation for 3 h. ^#^ Significant difference (*p* < 0.05). (**a**) CFU/mL values of *S. mutans* in washed PBS from the first incubation after being cultured on TSA using SP-SDS. (**b**) The OD_600_ values of *S. mutans* in culture medium after the second incubation for 13 h. (**c**) The CFU/mL values of *S. mutans* in culture medium after the second incubation for 13 h.

## Abbreviations

ATR-FTIR	attenuated total reflection-Fourier transform infrared
CFU	colony-forming units
CLSM	confocal laser scanning microscope
EG	ethylene glycol
F–M	Fairbrother–Mastin
HEMA	2-hydroxyethyl methacrylate
HGFs	human gingival fibroblasts
MMA	methyl methacrylate
MTT assay	3-(4,5-dimethylthiazol-2-yl)-2,5-diphenyl tetrazolium bromide assay
OD	optical density
OD_600_	OD value at 600 nm
PBS	phosphate buffered saline
PEGMA	polyethylene glycol methacrylate
pHEMA	poly(2-hydroxyethyl methacrylate)
PI	propidium iodide
PMMA	polymethyl methacrylate
PMMA-PEGMA	PMMA grafted with PEGMA
PMMA-pHEMA	PMMA grafted with pHEMA
QAMS	quaternary ammonium methacryloxy silicate
SP-SDS	single plate-serial dilution spotting
TSA	tryptic soy agar
TSB	tryptic soy broth

## Appendix A

Tests conducted by Moghayedi et al. on minimum bactericidal and inhibitory concentrations using a broth dilution assay have indicated the concentration threshold above which EG may act as a bactericidal agent against *E. coli*, with bacterial growth being completely inhibited within 4 h when the EG concentration is increased to 24% [36]. The dependence of bactericidal activity against *E. coli* on PEG concentration has also been reported previously [37]. The bactericidality may be related to the abnormal shape of bacterial cells placed in contact with concentrated EG and PEG [36,38]. This abnormality makes the cells tend to break and lysis occurs, as evidenced in phase-contrast microscopic observations of increased lysis in cells with greater changes in appearance [39]. Thus, for *E. coli*, the EG side chain of PMMA-pHEMA at the correct pHEMA surface concentrations may have caused PMMA-pHEMA to act, in addition to inhibiting adhesion through repulsion, as a bactericidal agent in this study, killing the bacteria (the center image of Figure 4) and causing lysis therein [36]. The lysis occurring in bacterial cultivation on PMMA-pHEMA produced lipopolysaccharides, which are a part of the outer membrane of Gram-negative bacteria, and released intracellular structures and compounds, including DNA.

These lysis fragments could act as biosurfactants [40]. For example, lipopolysaccharides are large molecules consisting of a hydrophobic, non-polar lipid (called Lipid A) at one end and a hydrophilic, polar polysaccharide (called *O*-specific polysaccharide) at the other. After cell lysis, their fragments could thus act as biosurfactants. The polar ends of the biosurfactants tended to be oriented inwards, because of dipole-dipole interaction, toward the hydrophilic PMMA-pHEMA surface. The non-polar ends then pointed outwards, changing the local areas from hydrophilic to hydrophobic, thus allowing more *E. coli* cell adhesion based on the biosurfactants. Accordingly, the biosurfactants generated by cell lysis reduced the hydrophilicity of the PMMA-pHEMA surface. The decrease in surface hydrophilicity caused by the production of biosurfactants such as lipopolysaccharides from the lysis of *E. coli* could alter the contact angle [40,41]. For example, coating titanium discs with different concentrations of *Porphyromonas gingivalis* lipopolysaccharides showed an increase in the average contact angle with higher concentrations, indicating the discs becoming less hydrophilic [42].

The less hydrophilic surface may have further interacted with *E. coli* cells that had become more hydrophobic because of the attachment of DNA, further enhancing cell adhesion to PMMA-pHEMA. Several studies have suggested that DNA in solution makes bacterial cells more hydrophobic [43,44]. DNA released from the lysis of *E. coli* by PMMA-pHEMA could thus attach to other *E. coli* cells, making them more hydrophobic. As the PMMA-pHEMA surface gradually became less hydrophilic with increasing amounts of biosurfactants being produced in bacterial cultivation, more DNA was released to the surface from the lysis of more cells. The interaction of the more hydrophobic cells with the increasingly less hydrophilic surface became more effective as more cell lysis occurred and more DNA was released. This attracted more *E. coli* cells to the surface, making them more hydrophobic, and thus further changing the hydrophilicity of the surface until an appropriate surface hydrophilicity was reached, at which point a large amount of *E. coli* cells could stably adhere, along with their fragments, to PMMA-pHEMA.

The biosurfactants produced from cell lysis could also have screened the repulsive interaction of PMMA-pHEMA with *E. coli* cells, allowing more *E. coli* cells to adhere to PMMA-pHEMA. Disruption of the enhanced adhesion of *E. coli* cells produced additional fragments that then acted as biosurfactants for further enhancement of cell adhesion. Overall, many *E. coli* cells adhered to PMMA-pHEMA based on the biosurfactants, with each *E. coli* layer sandwiched between two bi-layers of biosurfactants. The bi-layers were formed because once adhering to the PMMA-pHEMA surface, *E. coli* further interacted with the non-polar end of the biosurfactant near the surface, leaving the biosurfactant’s polar end to attract other biosurfactants and turning their non-polar ends outward, thus attracting additional *E. coli* cells for adhesion. The process continued locally, further enhancing the adhesion of *E. coli* by sandwiching each *E. coli* cell between two bi-layers of biosurfactant. This adhesion enhancement is hereafter termed the biosurfactant sandwich theory. The overall enhancement of cell adhesion thus led to a number of cells on PMMA-pHEMA as high as or higher than the amount on PMMA (Figure 5a). It should be noted that dental PMMA may be made of base materials containing negative ion powder, which, when amounting to more than 2 wt.%, demonstrate bactericidal activity as high as 99.1% against *E. coli* [45].

The adhesion of *E. coli* cells, based on the biosurfactants, to PMMA-pHEMA was considerably weak, however. Other than the dipole–dipole interaction mentioned in the main text, the biosurfactants generated by the lysis of *E. coli* cells may accumulate on the material surface through London dispersion interaction with nonuniform force plateaus [46]. The magnitude of this interaction was not strong but was of the order of osmotic pressure of the solution containing *E. coli* cells and their lysis fragments [47]. Because the accumulation occurred in solution, the space between interacting partners was not empty, but rather filled with water and possibly other solutes. The filling also weakened the biosurfactant-based adhesion of cells to PMMA-pHEMA. Thus, the adhered *E. coli* cells may have been relatively easily purged by PBS after the first incubation. The purge also removed the dead cells and their lysis fragments from the surface. Therefore, red spots identified in the corresponding CLSM image (the center image of Figure 4) were not as many as the biosurfactant sandwich theory suggests, which also differed largely from the large amount of adhered *E. coli* estimated from the washed PBS (Figure 5a). After the purge, only the *E. coli* cells that adhered to the “native” PMMA-pHEMA surface (as distinguished from the cells that adhered to PMMA-pHEMA between the biosurfactant bi-layers) before cell lysis remained for CLSM study and for the second incubation of cells. Thus, the amount of *E. coli* cells determined from the culture medium of the second incubation of PMMA-pHEMA (Figure 5b,c) was smaller than the amount determined from the PBS wash of the first incubation (Figure 5a). The difference in CFU/mL value obtained between the first and second incubations was thus an index of the change in surface hydrophilicity or the extent of the accumulation of biosurfactants and bacterial cells on the grafted PMMA surface during the first incubation.

Many factors determined the amount of *E. coli* adhered to the grafted PMMA and their interplay was extremely complex. The amount of adhered *E. coli* cells is determined by complex factors including the initial surface hydrophilicity, surface morphology, graft identity and concentration, graft orientation and bonding, and bacterial cell identity and concentration, as well as experimental conditions that affect fluid hydrodynamics, such as the rate at which bacterial cells are delivered to the grafted surface, the time the cells reside close to the surface, and the transport and residing of cell lysis fragments in close proximity to the surface. The complexity of the interplay may partially explain the controversial conclusions in the literature regarding the bactericidal activity of PEG-grafted devices. For example, antibacterial agents grafted to a material surface with different orientations may exhibit a range of antibacterial effect from none to large [48]. In fact, for PEGMA grafted on a material surface, the change in grafting orientation may change the grafted surface from bactericidal to cell-repellent through control of temperature within a small range around 35 °C [49]. A PMMA surface grafted with molecules such as PEGMA with PEG side chains longer than those of pHEMA may thus behave completely differently to a PMMA-pHEMA surface. However, knowledge of this interplay remains highly limited and requires further investigation.

Nevertheless, the much lower CFU/mL value (Figure 5a) for PMMA-PEGMA than for PMMA-pHEMA, compared with the higher value shown in Figure 5b,c, may be partly explained as follows. As discussed, more *E. coli* cells could adhere to PMMA-PEGMA than PMMA-pHEMA because PMMA-PEGMA was less hydrophilic. However, within a certain size range, the bactericidal activity of PEG (and, in this case, the PEG side chain) mainly increases with the size of PEG [37]. For example, PEG 400 showed bactericidal activity against *E. coli* at 100% concentration only, whereas PEG 1000 showed similar bactericidal activity at a concentration as low as 25%. Thus, at an appropriate surface concentration, PEGMA, which contains much longer PEG side chains than pHEMA, grafted on PMMA may kill and disrupt more adhered *E. coli* cells than pHEMA during the first incubation in which cell adhesion based on the biosurfactants occurs. Thus, other than the cells that could attach directly to the surface and adapt their configuration for survival, those sandwiched between surfactant bi-layers were more prone to be disrupted by the greater bactericidal activity of PMMA-PEGMA than PMMA-pHEMA. Thus, after the first incubation, fewer live *E. coli* cells could be washed out by PBS from PMMA-PEGMA than PMMA-pHEMA (Figure 5a). It should be indicated that lacking a lipid-like, non-polar end in its structure, the fragmented peptidoglycans generated by the lysis of *S. mutans* could not act as biosurfactants. As Gram-positive bacteria, *S. mutans* have a cell wall containing a large percentage of polyol phosphate polymers (called teichoic acid) that bear a strong negative charge. The large number of phosphate groups lining the wall’s surface aid the transport of peptidoglycan from within the cell to the outside. Consequently, the wall consists of a thick outer peptidoglycan layer and an inner cytoplasmic membrane, which is made of a lipid bi-layer. Because peptidoglycan forms approximately 90% of the dry weight of Gram-positive bacteria, the property of the PMMA-pHEMA and PEGMA surfaces in contact with *S. mutans* cells was altered mainly by peptidoglycans fragmented by cell lysis caused by the bactericidal activity of PMMA-pHEMA and PEGMA. As a polymer, peptidoglycan consists of sugars and amino acids, with a peptide chain of 3 to 5 amino acids attaching to *N*-acetylmuramic acid of the sugar component. They provide polar ends to the structure of the peptidoglycans fragmented by the lysis of *S. mutans*. Without non-polar ends, these fragments could not act as biosurfactants and could not reduce the hydrophilicity of the grafted PMMA as much as lipopolysaccharides did when they were produced by the lysis of *E. coli*. No biosurfactant-based adhesion to the PMMA-pHEMA and PEGMA surfaces was expected for *S. mutans*, even though the property of these surfaces in contact with *S. mutans* cells may still be altered by peptidoglycans fragmented by cell lysis caused by the bactericidal activity of PMMA-pHEMA and PEGMA.
